# Comparison of the power and type 1 error of total score models for drug effect detection in clinical trials

**DOI:** 10.1007/s10928-024-09949-0

**Published:** 2024-12-10

**Authors:** Elham Haem, Mats O. Karlsson, Sebastian Ueckert

**Affiliations:** 1 Department of Biostatistics, School of Medicine, Shiraz University of Medical Sciences, Shiraz, Iran; 2 Pharmacometrics Research Group, Department of Pharmacy, Uppsala University, Uppsala, Sweden

**Keywords:** Total score data, Bounded integer model, Coarsened grid model, IRT-informed total score analysis

## Abstract

Composite scale data consists of numerous categorical questions/items that are often summed as a total score and are commonly utilized as primary endpoints in clinical trials. These endpoints are conceptually discrete and constrained by nature. Item response theory (IRT) is a powerful approach for detecting drug effects in composite scale data from clinical trials, but estimating all parameters requires a large sample size and all item information, which may not be available. Therefore, total score models are often utilized. The most popular total score models are continuous variable (CV) models, but this strategy establishes assumptions that go against the integer nature, and typically also the bounded nature, of data. Bounded integer (BI) and Coarsened grid (CG) models respect the nature of the data. However, their power to detect drug effects has not been as thoroughly studied in clinical trials. When an IRT model is accessible, IRT-informed models (I-BI and I-CV) are promising methods in which the mean and variability of the total score at any position are extracted from the existing IRT model. In this study, total score data were simulated from the MDS-UPDRS motor subscale. Then, the power, type 1 error, and treatment effect bias of six total score models for detecting drug effects in clinical trials were explored. Further, it was investigated how the power, type 1 of error, and treatment effect bias for the I-BI and I-CV models were affected by mis-specified item information from the IRT model. The I-BI model demonstrated the highest statistical power, maintained an acceptable Type I error rate, and exhibited minimal bias, approaching zero. Following that, the I-CV, BI, and CG with Czado transformation (CG_Czado) models provided the maximum power. However, the CG_Czado model had inflated type 1 error under low sample size scenarios in each arm of clinical trials. The CG model among total score models displayed the lowest power and the most inflated type 1 error. Therefore, the results favor the I-BI model when an IRT model is available; otherwise, the BI model.

**Supplementary Information:**

The online version contains supplementary material available at 10.1007/s10928-024-09949-0.

## Introduction

Composite scales, which provide a measure of disease severity, are utilized to quantify many clinical study endpoints. A composite scale is made up of a number of assessments or questions that add up to a total score. Modeling this complex scale-based data can be done in a number of ways.

Item response theory (IRT) models have been used in a pharmacometric framework to analyze composite scales [1–3]. These models seek to explain the relationship between a patient’s disability and their responses to items on the assessment scale. Since the IRT approach uses item level data and all the information collected, it is the most informative way to analyze composite scale data. Using one or more latent variables, this approach estimates item characteristic curves (ICCs) for each item and incorporates correlation between items. In simulated trials, the IRT-based model regularly outperforms the sum of item scores analysis in terms of power on drug effect detection [4].

This approach, however, is complex to develop, requires a large data set to estimate all item parameters, and takes a long time to estimate. The IRT-based model, in particular, necessitates item-level data, which may not be accessible [5]. Thus, it is interesting to study the total score of a composite scale. Total score data can be described using several dedicated methods, including continuous variable (CV), bounded integer (BI) [6], coarsened grid (CG) [7], and IRT-informed models [8].

The continuous variable (CV) model, which treats the outcomes as a continuous variable while the underlying data is categorical or integer, is one of the most popular total score models. CV models are easy to implement, but they can be problematic, especially at scale boundaries, where the residual error can cause predictions to go beyond the expected range [6]. BI models respect the boundaries and data nature. They are defined by a latent grid of quintiles of the normal distribution. Each subject’s location in that latent grid is described both in terms of its mean and variance over time [9]. When compared to the CV model, the BI approach generally provided a better description of numerous different scales [10]. On the other hand, the power and type 1 error of BI models were compared with traditional methods such as CV and ordered categorical (OC) approach in drug effect detection and resulted in a similar performance of BI models. However, they displayed more type 1 error than OC models [10].

The coarsened grid (CG) model, which uses logit transformation for bounded outcome scores, is an alternate approach. It assumes a latent continuous variable of the bounded outcome score values on the interval [0,1] which follows a logit-normal distribution [7]. Moreover, an additional complex link function from the Czado family can be used in the CG model to modify both tails of the standard logit transformation and it was demonstrated that CG with Czado transformation produced promising results for one data set [7].

IRT-informed models have recently been proposed as a method for describing total score data when there exists an IRT model for the same composite scale. Since the variability at the limits of bounded data is lower, a homoscedastic error, which is frequently used in CV models, is not the best technique to explain the variability. Instead, item characteristic curves can be used to calculate the mean and variability at each latent variable value in an IRT model. As a result, an IRT model can yield the expected variability at any total score value. In addition, this technique can be used in BI models. IRT-informed BI (I-BI) models allow for a direct translation between the IRT model’s latent variable and the expected mean and SD calculated on the Z score scale [8].

The precision and accuracy of IRT-informed models and other total score models were assessed in a simulated phase 3 clinical trial setting in Parkinson’s disease. The IRT-informed models (I-CV and I-BI) were found to have the best fit as well as the best performance on external data. Furthermore, the IRT link functions enabled the retrieval of IRT parameters with high precision and low bias, particularly in the I-BI model [5]. I-CV and I-BI models were investigated on both simulated and real data, in another study. Both CV and BI models were shown to improve in fit by IRT-informed disease progression, which offers precise and accurate corresponding latent variable parameters [8].

There is no comprehensive literature that evaluates the power to detect a drug effect, the corresponding type 1 error, and treatment effect bias across a broad range of total score models. Although there exists literature that has evaluated the performance of total score models [5, 8], the current study comprehensively assessed different analysis methods, including the original IRT model, IRT-informed total score models, and non-IRT-informed total score models covering CG and CG_Czado not limited to CV and BI.

In the present study, an IRT model was used to simulate data in phase 3 clinical trial setting of Parkinson’s disease to compare the power, type 1 error, and treatment effect bias of CV, BI, CG, CG with Czado transformation as well as the true IRT model. Additionally, the impact of mis-specified item information from the IRT model on the power, type 1 error, and treatment effect bias of the IRT, I-CV, and I-BI models was examined for the first time.

## Methods

### Simulation model & design

A previously published IRT model [4] for MDS-UPDRS motor was used to simulate data from a clinical trial setting in Parkinson’s disease over a two-year period. The concept of an IRT model is described below.

Item Response Theory (IRT), is a statistical framework that utilizes mathematical models to elucidate the relationship between an individual’s responses to items on an assessment scale and an underlying, unobservable trait—here, the patient’s level of disability. One of the popular functional forms of the IRT models for ordered categorical data are

$$\:p\left({y}_{ij}\ge\:k\right)=\:\frac{{e}^{{a}_{j}\:.\:\:\left({D}_{i}-\:{b}_{jk}\right)}}{1+\:{e}^{{a}_{j}\:.\:\:\left({D}_{i}-\:{b}_{jk}\right)}\:}$$

$$\:p\left({y}_{ij}=k\right)=\:p\left({y}_{ij}\ge\:k\right)-\:p\left({y}_{ij}\ge\:k+1\right)$$

where $\:p\left({y}_{ij}\ge\:k\right)$ represents the probability that the observed score for item j and patient i is greater than or equal to k, the number of response categories. $\:{D}_{i}$ represents the unobserved IRT-based disability level of patient i, which varies as a function of time and drug effect during clinical trials. Additionally, $\:{a}_{j}$ and $\:{b}_{jk}$ represent the item discrimination parameter and the item difficulty parameters, respectively, with the latter being one less than the number of response categories. The discrimination parameter indicates how well an item distinguishes between individuals with varying levels of disability. A higher discrimination value suggests that the item is more effective at differentiating between patients with different levels of disability. The difficulty parameter is the disability level at which a patient has a 50% chance of responding at or above a certain score. Items with higher difficulty parameters require a higher level of disability for patients to respond with higher scores, meaning individuals with more severe disability are more likely to select higher response options [3].

In the first step of this simulation study, the parameter estimates for discrimination and difficulties from the MDS-UPDRS motor data in the Buatois et al. [4] study were used to simulate data, as detailed in the supplementary materials. In the next step, a linear model was considered on the unobserved IRT disability of patient $\:{D}_{i}$ over time; see the following:

$$\:{D}_{i}\left(t\right)=\:{D}_{i0}+\:{slope}_{i\:TRT}\:\times\:t\:,\:\:\:TRT=\text{1,2}\:\:\:\:\:\:\:\:$$

where$\:\:{D}_{i}\left(t\right)$ is the unobserved IRT disability of patient i, $\:{D}_{i0}$ characterizes the disability at baseline as a random parameter, and also $\:{slope}_{i\:TRT}$ is a subject specific random parameter that characterizes the rate of disability change for placebo (TRT = 1) and treatment (TRT = 2) arms in the clinical trial. Random effects in Eq. (1) have the following functional form:

$$\:{D}_{i0}={\theta\:}_{0}^{base}+\:{\eta\:}_{ibase}\:\:\:\:\:\:\:\:\:\:\:\:\:\:\:\:\:\:\:\:\:\:\:\:\:\:\:\:\:\:\:\:\:\:\:\:\:\:\:\:\:\:\:\:\:\:\:\:\:\:\:\:\:\:\:\:\:\:\:\:\:\:\:\:\:\:\:\:\:\:\:\:\:\:\:\:\:\:\:\:\:\:\:\:\:\:\:\:\:\:\:\:\:\:\:\:\:\:\:\:\:\:$$

$$\:{slope}_{i\:TRT}={\theta\:}_{1TRT}^{slope}+{\eta\:}_{iTRT}\:\:,\:TRT=\text{1,2}\:\:\:\:\:\:\:\:\:\:\:\:\:\:\:\:\:\:\:\:\:\:\:\:\:\:\:\:\:\:\:\:\:\:\:\:\:\:\:\:\:\:\:\:\:\:\:\:\:\:\:\:\:\:\:\:\:\:\:\:\:\:\:\:\:\:\:\:$$

where $\:{\theta\:}_{0}^{base}$, $\:{\theta\:}_{1TRT}^{slope}$ are fixed effects (set to 0.1, 0.451, and 0.351), $\:{\eta\:}_{ibase}$ and $\:{\eta\:}_{iTRT}$ are normally distributed random variables causing inter-individual variability with variances $\:{\omega\:}_{base\:}^{2}=1,\:{\omega\:}_{TRT}^{2}=0.09$, respectively. These selected values represent an approximate 30% drug effect size and capture realistic levels of variability commonly observed in clinical trials.

In three different scenarios, 25, 50, and 75 subjects were simulated in each arm and followed across 5 visits at baseline, 0.5, 1, 1.5, and 2 years. In each scenario, the simulation was performed under two settings: (i) simulation with a drug effect to evaluate power and (ii) simulation without a drug effect to evaluate the type 1 error. For the power evaluation, a drug effect was chosen to achieve approximately 80% power with the true IRT model. In both settings, 1000 replicates were generated to calculate the power and type 1 error. Further, treatment effect bias was calculated in all scenarios.

### Estimation models

The simulated total scores were derived from the sum of individual item scores. Six different models for total score data, as well as the true IRT model (in which item parameters were fixed to the simulation values), were compared in this study: CV, BI, CG, CG with Czado transformation (CG_Czado) as well as IRT-informed CV and BI models (I-CV and I-BI). Furthermore, in a separate section, the effect of mis-specified item parameters was investigated on the power, type 1 error, and treatment effect bias of IRT, I-BI, and I-CV models. In this section, difficulty and discrimination parameters were purposely changed in the IRT-informed functions in order to evaluate the effect of the mis-specification of IRT-informed functions on the IRT-informed models.

The total score models mentioned above are described here.

### Continuous variable models

The functional form for the standard CV model with homoscedastic error follows:

$$\:{Y}_{ij}=f\left(\varTheta\:,\:{\eta\:}_{i},\:{t}_{ij},{TRT}_{i}\right)+\:{\epsilon\:}_{ij}$$

$$\:f\left(\varTheta\:,\:{\eta\:}_{i},\:{t}_{ij},{TRT}_{i}\right)={base}_{i}+{slope}_{iTRT}\times\:{t}_{ij}$$

$$\:{\:\:\:\:\:\:\:\:\:\:\:\:\:\:\:\:\:\:\:\:\:\:\:\:\:\:\:\:\:\:\:\:\:\:base}_{i}={\theta\:}_{1}$$+$$\:{\eta\:}_{i1}$$ & $$\:{slope}_{iTRT}={\theta\:}_{2TRT}+{\eta\:}_{iTRT}$$

$$\:\eta {\:_i}\: \sim N\left( {0,\omega {\:^2}} \right)$$

$$\:\varepsilon {\:_{ij}} \sim N\left( {0,\sigma {\:^2}} \right)$$

where $\:{Y}_{ij}$ denotes the observation j for subject i at time $\:{t}_{ij}$, $\:\varTheta\:$ the fixed effect parameters, $\:{\eta\:}_{i}$ the random effects of the inter-individual, $\:{TRT}_{i}$ the type of drug (treatment or placebo), $\:{\epsilon\:}_{ij}$ the residual unexplained variability (RUV), $\:{\omega\:}^{2}$ the variance of inter-individual variability and $\:{\sigma\:}^{2}$ the variance of the RUV [5].

### IRT-informed CV model

The fully IRT-informed CV model (I-CV) is defined as:

$$\:{Y}_{ij}=p{n}_{1}\left({\psi\:}_{ij}\right)+\:{\epsilon\:}_{ij}\:.\:p{n}_{2}\left({\psi\:}_{ij}\right)$$

$$\:{\psi\:}_{ij}=f\left(\theta\:,{\eta\:}_{i},\:{t}_{ij},{TRT}_{i}\right)$$

$$\:\eta {\:_i}\: \sim N\left( {0,\omega {\:^2}} \right)$$

$$\:\varepsilon {\:_{ij}} \sim N\left( {0,\sigma {\:^2}} \right))$$

where $\:{\psi\:}_{ij}$ is a latent variable described by the linear function $\:f\left(.\right)$, which characterizes the rate of disability change for placebo and treatment arms, and $\:p{n}_{1}$as well as $\:p{n}_{2}$ are predetermined

polynomials [8]. The other variables bear the same definition as the CV models.

### Bounded integer models

The BI model is a discrete data model presuming a latent underlying continuous variable of the observed total score. In this approach, for a given latent variable U, a standardized transformed total score (STS) on the closed interval [0, 1] is defined. Let n be the number of observed total score values. Thus STS takes the form of k/n, where k = 0,…, n. The latent variable U is related to the STS through:

$$\:STS=\frac{K}{n\:}\:if\:and\:only\:if\:{a}_{k}\le\:U\le\:{a}_{k+1}\:,\:\:\:\:\:\:\:\:\:\:\:for\:k=0,\dots\:,n$$

where $\:{a}_{k}=\frac{\left(k-0.5\right)}{n}$ and $\:{a}_{k+1}=\frac{\left(k+0.5\right)}{n}$, with $\:{a}_{0}=0$ and $\:{a}_{n+1}=1$.

Further, we define p, the individual prediction:

$$\:probit\left(U\right)=p$$

where p follows a normal distribution with the mean of the linear function $\:f\left(.\right)$, which was defined in the previous section, and $\sigma$as the variance of the latent variable.

The probability of individual i observing a category STS = k/n is defined through:

$$\begin{aligned}\:{P}_{i,j}\left(k\right) & =\varphi\:\left(\frac{{Z}_{\frac{k}{n}}-f\left(\theta\:,{\eta\:}_{i},\:{t}_{ij},{TRT}_{i}\right)\:}{\sigma}\right)\\& \quad -\varphi\:\left(\frac{{Z}_{\frac{k-1}{n}}-f\left(\theta\:,{\eta\:}_{i},\:{t}_{ij},{TRT}_{i}\right)\:}{\sigma}\right)\end{aligned}$$

With special cases for the first and last categories, the previous equation collapses into:

$$\:{P}_{i,j}\left(1\right)=\varphi\:\left(\frac{{Z}_{\frac{1}{n}}-f\left(\theta\:,{\eta\:}_{i},\:{t}_{ij},{TRT}_{i}\right)\:}{\sigma}\right)$$

$$\:{P}_{i,j}\left(n\right)=1-\varphi\:\left(\frac{{Z}_{\frac{n-1}{n}}-f\left(\theta\:,{\eta\:}_{i},\:{t}_{ij},{TRT}_{i}\right)\:}{\sigma}\right)$$

where φ is the cumulative distribution function of the normal distribution and $\:{Z}_{\frac{k}{n}}$and $\:{Z}_{\frac{\left(k-1\right)}{n}}\:$are the cut points between categories k and k-1 defined through the probit function [6].

### IRT-informed BI model

The fully IRT-informed BI model (I-BI) is defined as:

$$\:{P}_{i,j}\left(k\right)=\varphi\:\left(\frac{{Z}_{\frac{k}{n}}-p{n}_{3}\:\left({\psi\:}_{ij}\right)}{p{n}_{4}\:\left({\psi\:}_{ij}\right)}\right)-\varphi\:\left(\frac{{Z}_{\frac{k-1}{n}}-p{n}_{3}\:\left({\psi\:}_{ij}\right)\:}{p{n}_{4}\:\left({\psi\:}_{ij}\right)}\right)$$

Where $\:{\psi\:}_{ij}$ is considered the same as the I-CV model, however, $\:p{n}_{3}$ and $\:p{n}_{4}$ are predetermined polynomials that are distinct from polynomials in the I-CV model [8].

### Coarsened grid model

A CG approach is another discrete analysis resembling BI which presumes a latent variable U on the open interval (0, 1). The only distinction with BI is that a logit-normal distribution is assumed for the underlying latent variable:

$$\:logit\left( U \right) \sim N\left( {f\left( {\theta \:,\eta {\:_i},\:{t_{ij}},TR{T_i}} \right),\sigma {\:^2}\:} \right)$$

The probability of observing a category STS = k/n is defined as:

$$\begin{aligned}\:P\left({Y}_{ij} = k\right) &  = \varphi\:\left(\frac{{Z}_{k}^{\left(u\right)}-f\left(\theta\:,{\eta\:}_{i},\:{t}_{ij},{TRT}_{i}\right)\:}{\sigma}\right) \\& \quad -\varphi\:\left(\frac{{Z}_{k}^{\left(l\right)}-f\left(\theta\:,{\eta\:}_{i},\:{t}_{ij},{TRT}_{i}\right)\:}{\sigma}\right)\end{aligned}$$

Where $\:{Z}_{k}^{\left(l\right)}=logit\left({a}_{k}\right)$, $\:{Z}_{k}^{\left(u\right)}=logit\left({a}_{k+1}\right)$, and $\:f\left(.\right)$ is a linear function as same as previous sections.

An additional flexible transformation like Czado [7], can be used to accommodate skewed data distributions with two parameters $\:\left({\lambda\:}_{1},{\lambda\:}_{2}\:\right)$ as below:

$$\:h\left(x,{\lambda\:}_{1},{\lambda\:}_{2}\:\right)=\left\{\begin{array}{c}\frac{{\left(x+1\right)}^{{\lambda\:}_{1}}-1}{{\lambda\:}_{1}},\:\:x\ge\:0\\\:-\frac{{\left(-x+1\right)}^{{\lambda\:}_{2}}-1}{{\lambda\:}_{2}},\:\:x<0\end{array}\right.$$

where $\:{\lambda\:}_{1},\:\text{a}\text{n}\text{d}\:{\lambda\:}_{2}$ are parameters to be estimated [7, 11].

### Software

The total score models were generated through the piraid package in R software. Afterwards the simulation and the estimation of the models were performed in NONMEM version 7.4 (ICON Development Solutions, Ellicott City, MD) with the help of PsN version 4.9 [12]. The stochastic approximation expectation maximization (SAEM) algorithm was used as an estimation algorithm followed by importance sampling to evaluate the log-likelihood and compare the OFV between models. The statistical software RStudio1.1.383 using R 3.6.1 and ggplot2 3.3.3 were implemented to generate statistical analysis and graphical presentations.

**Fig. 1:**
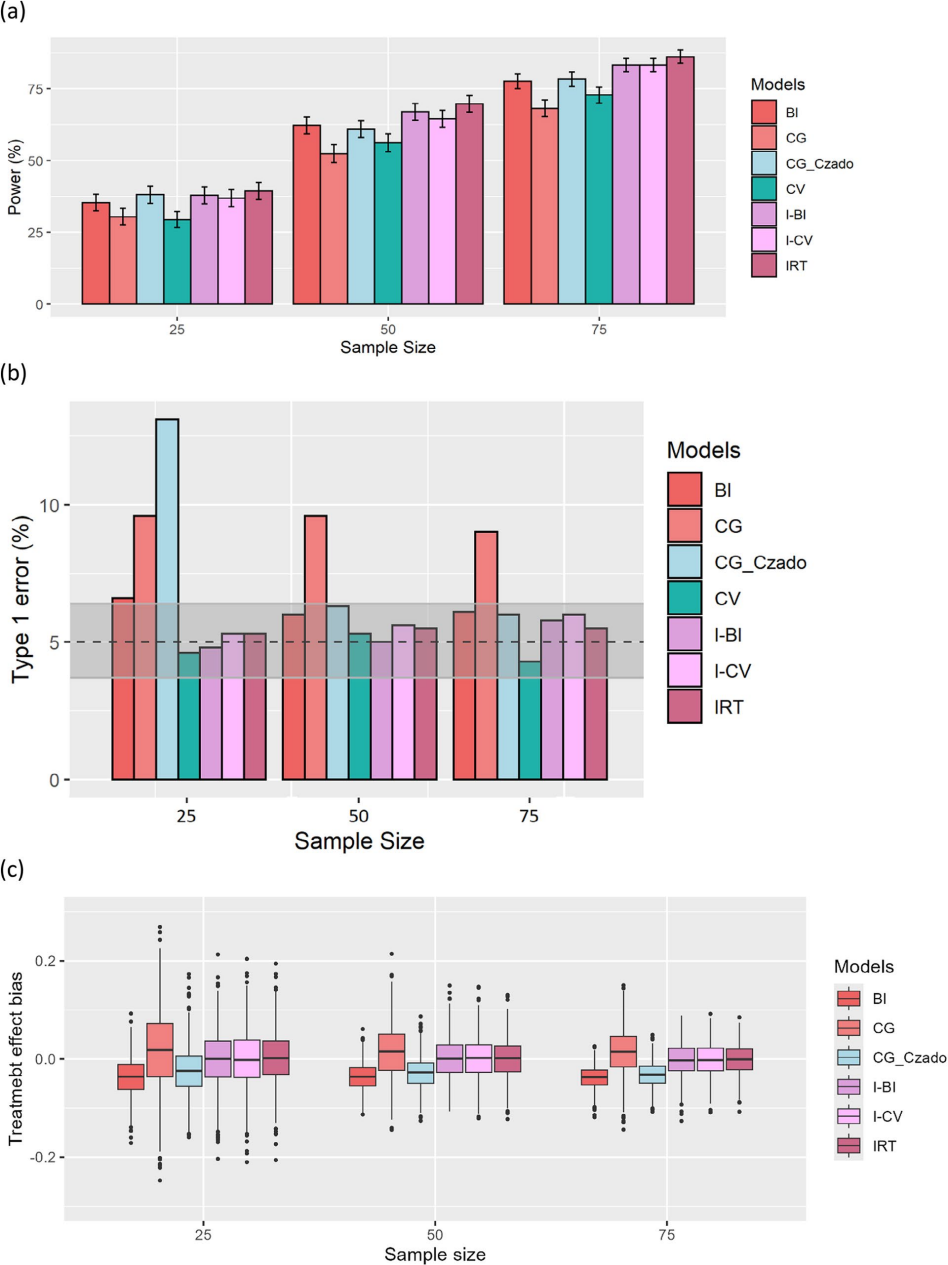
(**a**) Power and its 95% confidence interval, (**b**) associated type 1 error and its 95% confidence interval, and (**c**) bias of treatment effect estimates of the true IRT models and total score models for different number of patients in each arm of clinical trials

## Result

Model convergence was not a problem for any of the models implemented in NONMEM. Throughout the simulation in various scenarios, no failed terminations were observed. Findings in this simulation study are presented in two parts: total score models and miss-specified IRT-informed models.

### Total score models

Figure 1. a displays the power of the true IRT, CV, I-CV, BI, I-BI, CG, and CG_Czado models under various numbers of subjects in each arm of the simulated clinical trial to detect a drug effect. As anticipated, the IRT model had the maximum power across all sample sizes, although there was no discernible difference between it and the I-BI, I-CV, and CG_Czado models under 25 participants in each arm.

It is evident that IRT-informed models (I-BI and I-CV) provided higher power in comparison to other total score models, although the CG_Czado model had the same power under the 25-subject scenario. It is noteworthy that when IRT-informed models were disregarded, the BI and CG_Czado models offered greater power than other models. Moreover, the CG and CV models had the lowest power to detect drug effects, respectively.

Figure 1. b shows type 1 error of the true IRT, CV, I-CV, BI, I-BI, CG, and CG_Czado models in different scenarios. While the true IRT, I-BI, I-CV, and CV models maintained type I error rates around the expected 5%, the CG model consistently exhibited inflated type I errors across all scenarios. Notably, the CG_Czado model displayed inflated type I errors only at a sample size of *N* = 25, with acceptable error rates falling within the confidence intervals at *N* = 50 and *N* = 75. In contrast, the BI model showed a slightly higher type I error rate compared to the IRT-informed and CV models; however, it remained significantly lower than the CG model. Additionally, the BI model demonstrated a comparable type 1 error rate to the CG_Czado model, except at low sample sizes, where the CG_Czado model experienced inflated type 1 errors.

Figure 1. c presents the bias in treatment effect estimates for the true IRT and total score models, excluding the CV model. The CV model exhibited substantial bias in its treatment effect estimates, which obscured the differences between the other models. As a result, the box plot for the CV model was omitted from the figure to ensure a clearer comparison among the remaining models. The true IRT and IRT-informed models showed minimal bias, with a noticeable decrease in the variability of the box plots as the sample size increased. Additionally, the median of the CG_Czado model was lower than that of both the CG and BI models.

### Mis-specified IRT-informed models

**Fig. 2:**
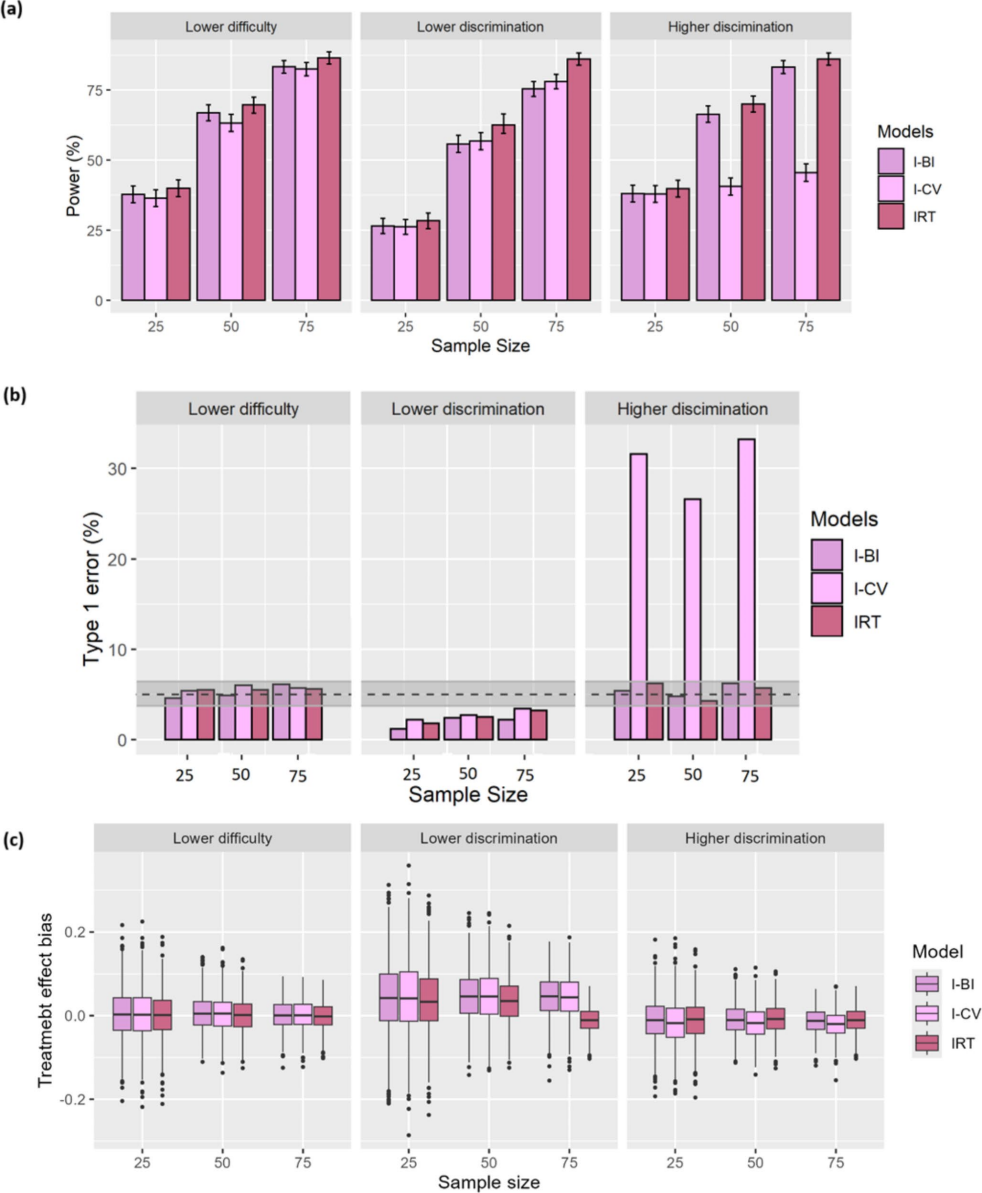
(**a**) Power and its confidence interval, (**b**) type 1 error and its 95% confidence interval, and (**c**) bias of treatment effect estimates of IRT, I-BI, and I-CV models for different numbers of patients in each arm of clinical trials

In the second part, the power, type 1 error, and bias of treatment effect estimate for mis-specified IRT and IRT-informed models (I-BI and I-CV) were assessed when difficulty or discrimination parameters were purposefully altered. Figure 2. a and Fig. 2. b, depict the power and type 1 error along with its confidence interval for mis-specified IRT, I-CV, and I-BI models in the three different panels. When difficultyparameters were reduced (all difficulty values minus 1), the power and Type 1 error (Fig. 2. b) of the mis-specified IRT, I-BI, and I-CV models did not significantly change compared to the correctly specified models shown in Fig. 1. a and Fig. 1. b.

In Fig. 2. a, when discrimination parameters decreased (all parameter values were halved), the power of the mis-specified models dramatically decreased in comparison with the correctly specified models (Fig. 1. a), but the IRT model when sample size is 75. Likewise, Fig. 2. b shows that Type 1 error for miss-specified IRT, I-CV, and I-BI models decreased.

In comparison with the correctly specified models, when discrimination parameters increased (the values were multiplied by 1.5), the power of mis-specified IRT and I-BI models did not remarkably change. However, the power of mis-specified I-CV was significantly reduced (Fig. 2. a). Further, Type 1 errors of mis-specified IRT and I-BI models were not notably altered, but mis-specified I-CV showed severely inflated type 1 error (Fig. 2. b).

Figure 2. c illustrates the bias in treatment effect estimates for the mis-specified IRT, I-CV, and I-BI models across the three panels. As anticipated, the mis-specified IRT model exhibited minimal bias in the treatment effect estimates, with values close to zero. Additionally, the mis-specified IRT-informed models generally demonstrated small biases, except in cases where the discrimination parameters were reduced.

## Discussion

The current study compared the power, type 1 error, and treatment effect bias of total score models for various numbers of subjects in each arm of simulated clinical trials. IRT-informed models (I-BI and I-CV) provided more power and reasonable type 1 error amongst total score models. The IRT-informed functions formally connect the IRT and total score models. As a result, the total score models are enhanced because the expected score variability is more accurately described. Since the functions allow the SD to vary with the severity of the disease and follow the nature of total score data, they improve the fit while they do not need any additional parameters. The functions additionally might enable the retrieval of the longitudinal parameters of an IRT model with a total score model, which is advantageous to detect a potential drug effect in a clinical trial. IRT-informed models were further investigated in terms of predictive performance and precision [5, 8]. IRT-informed functions improved both CV and BI models in fit [8], while the I-BI model’s parameter estimates were more precise than those of the I-CV model [5]. Our findings suggest that, although Fig. 1 presents a similar visual depiction of power and type 1 error rates for the I-BI and I-CV models, quantitative analysis shows that the I-BI model consistently demonstrates higher statistical power and a lower type 1 error rate across different sample sizes. This is especially notable in smaller clinical trials. Cited studies support these conclusions. Regarding treatment effect estimates, both models exhibited minimal bias across all scenarios. Therefore, while the I-CV model is effective for detecting drug effects, the I-BI model is generally more advantageous, particularly in smaller clinical trials.

A disadvantage of IRT-informed models is that they require an existing IRT model for the scale in a specified population, which may not be available. Given that IRT models require a large sample size to estimate parameters of difficulty and discrimination, they are infrequent, especially in developing countries where access to large amounts of data in clinical trials is prohibitively expensive and time-consuming. Therefore, it is essential to investigate alternative total score models, which are not IRT-informed, to advise modelers on the best model. Our research found that both the BI and CG_Czado models demonstrated comparable levels of statistical power, which were significantly higher than those of the CV and CG models. Additionally, while the median bias in treatment effect estimates for the CG_Czado model was slightly lower than that of the BI model, it exhibited greater variability, particularly with smaller sample sizes. Notably, the CG_Czado model showed an inflated type I error rate when sample sizes were small (25 subjects per group). When IRT-informed models are not feasible, both BI and CG_Czado models perform well with sample sizes of 50 and 75 in each group. The BI model seems to be the best choice for analyzing total score data when dealing with low sample sizes.

The BI model is a promising technique in analyzing total score data which has been recently focused on. A simulation-based study comparing the BI and CV models by AIC criteria concluded that the BI model was a better fit than the CV model [5]. In addition, the BI and CV models were implemented to characterize six real data sets from various clinical areas and scales. Except for one data set, it was demonstrated that the fit (likelihood) was better for the BI model than for the analogous CV model which supports the obtained results in the present study [6]. However, similar power and associated type 1 error between BI modeling and the CV in detecting drug effects were reported in a simulation study [10]. One of the reasons for this discrepancy could be the implementation of an improved version of the BI in the present study, while it was not used in the cited study. An improved implementation based on an approximation of the logarithm of the error function for BI modeling was suggested by Ueckert et al. [9]. It was found that when the within-subject variability was low, and parameter estimation was carried out using the Laplace method, the improved algorithm produced more accurate and less biased parameter estimates [9]. Thus, the precise and less biased parameter estimations in improved numerical stability for the BI model may lead to more power in detecting drug effects in clinical trials.

Another reason for the different results between the current work and the cited simulation-based study [10] could be the different number of trial replicates in the stochastic simulation and estimation (SSE) procedure to assess power and type 1 error in detecting drug effects. In the current study, the results were obtained based on 1000 replicate trials in the SSE procedure, while only 500 replicates were used in the cited simulation study. The number of replicate simulations depends on the complexity of the models. It appears that in simulation procedures where data are generated based on a complicated model such as an IRT model, only 500 replicate trials are not able to catch variability and result in stable power and type 1 error. In order to ensure enough replicate simulations, the first 500 replicate trials in the SSE procedure were used to calculate the power and the associated type 1 error of total score models. The SSE procedure was replicated three times with differentseeds to ensure the results obtained. However, this amount of replicate trials yielded unstable results including significantly different patterns in power and type 1 error of total score models in the three different SSE procedures. Therefore, three different SSE procedures with 1000 replicate trials and different seeds were run to evaluate the stability of the obtained results. Insignificant differences were observed amongst power and type 1 error of total score models in the three separate SSE procedures. Thus, in the present study, all the results in different scenarios were reported based on 1000 replicate simulations.

The CG and CG_Czado models also were investigated in this work. As mentioned before, the CG_Czado offered nearly a similar power to the BI model while suffering from severely inflated type 1 error under the 25 sample size of the trial. Moreover, the CG model was the worst among total score models with respect to type 1 error. The distribution of total score data from composite scales is often J or U-shaped [7]. The CG_Czado model using additional flexible transformation is proposed when data distribution appears skewed, while the CG is an appropriate model facing symmetric distribution [11]. In the current study, data was simulated based on a published IRT model in a clinical trial on Parkinson [4]. Thus, the simulated responses appear skewed. Therefore, the non-symmetric data distribution can justify the CG model’s performance. The CG-Czado model, which utilizes additional transformation parameters, encountered difficulties in accurately estimating model parameters when applied to small sample sizes, potentially resulting in unstable model performance.

Mis-specified IRT, I-BI, and I-CV models likewise were investigated in various scenarios. As discrimination parameters increase, the mis-specified I-CV model shows a significant drop in power and a sharp rise in type I error. Additionally, type 1 error of mis-specified models is lower than 5% and out of its confidence interval when there is a decrease in discrimination parameters. This decrease also introduces bias in treatment effect estimates, though no substantial bias is observed in other scenarios. It appears that when discrimination parameters are mis-specified, the inference under these IRT-informed models is impaired, and one needs to be aware of when using these types of models. However, when there are mis-specified difficulty parameters, it appears to influence less power, type 1 error, and treatment effect bias. Thus, in the absence of an IRT model for the scale in a particular population, analyzers need to be cautious in the application of IRT-informed models.

One limitation of our study is the focus on simulated data from a specific IRT model developed in the context of Parkinson’s disease (PD) and applied to a particular rating scale. Our conclusions on the total score models are based on a single simulation setting and may not necessarily extend to other diseases or alternative IRT model formulations. Future research should explore additional examples from different clinical contexts and use varied IRT models to evaluate the robustness of our conclusions. Expanding the scope of the simulation study to include different disease areas and rating scales would provide a more comprehensive understanding of the performance of these methods. Therefore, while our results offer valuable insights, they should be interpreted with caution and not overgeneralized beyond the specific context studied.

## Conclusion

There are several approaches to model total scores acquired from composite scales in clinical trials; the current study compared the power, type 1 error, and bias of treatment effect estimates for each method and provided guidance on when to select a particular method. When an IRT model for the scale is available, the I-BI appears to be the best option with the highest power, controlled type 1 error, and minimal treatment effect bias. In the absence of an IRT model for the scale, BI modeling appears favorable.

## Electronic supplementary material

Below is the link to the electronic supplementary material.

Supplementary Material 1

## Data Availability

No datasets were generated or analysed during the current study.
