# Supplementary material for: Comparison of the power and type 1 error of total score models for drug effect detection in clinical trials
Source: J Pharmacokinet Pharmacodyn. 2024 Dec 10;52(1):4. doi: 10.1007/s10928-024-09949-0 (PMC11632077; doi:10.1007/s10928-024-09949-0)
Supplement: Supplementary file 1 — Supplementary Material 1 [file 10928_2024_9949_MOESM1_ESM.docx]

**Supplemental Table 1:** Discrimination and Difficulty Parameter Values for MDS-UPDRS Motor Subscale Used in Data Simulation, including Items with Varying Numbers of Categories.

| items | Discrimination | Difficulty 1 | Difficulty 2 | Difficulty 3 |
| --- | --- | --- | --- | --- |
| Speech | 1.14 | 1.26 | 1.18 |  |
| Saliva and drooling | 1.11 | 1.22 | 0.724 | 1.11 |
| Chewing and swallowing | 1.04 | 2.46 |  |  |
| Eating tasks | 1.42 | 1.16 | 1.95 |  |
| Dressing | 2.01 | 0.867 | 1.53 |  |
| Hygiene | 1.65 | 1.36 |  |  |
| Handwriting | 1.18 | 0.263 | 1.46 | 1.34 |
| Doing hobbies and other activities | 1.39 | 1 | 1.46 |  |
| Turning in bed | 1.24 | 1.43 | 3.78 |  |
| Getting out of bed | 1.35 | 0.82 | 1.95 |  |
| Walking and balance | 1.38 | 0.945 | 2.32 |  |
| Freezing | 1.67 | 2.53 |  |  |
| Speech | 1.69 | 0.758 | 1.88 |  |
| Facial expression | 2.18 | -0.206 | 1.48 | 1.52 |
| Rigidity– Neck | 1.97 | 0.741 | 0.869 | 1.64 |
| Rigidity– Rigidity-RUE | 1.83 | -0.483 | 1.15 | 1.86 |
| Rigidity– Rigidity-LUE | 1.66 | 0.881 | 1.26 |  |
| Rigidity– Rigidity-RLE | 1.62 | 0.54 | 0.978 | 1.59 |
| Rigidity– Rigidity-LLE | 1.83 | 1.42 | 0.916 |  |
| Finger tapping–right hand | 2.25 | -0.65 | 1.31 | 1.1 |
| Finger tapping– left hand | 1.89 | 0.718 | 1.2 | 1.28 |
| Hand movements–right hand | 2.37 | -0.215 | 1.18 | 1.08 |
| Hand movements– left hand | 2.05 | 0.98 | 1.15 |  |
| Pronation- supination movements– right hand | 1.98 | -0.251 | 1.22 | 1.21 |
| Pronation- supination movements– left hand | 2.01 | 1.09 | 1.19 |  |
| Toe tapping– right foot | 1.99 | -0.334 | 1.35 | 1.24 |
| Toe tapping– left foot | 1.45 | 0.89 | 1.53 | 1.67 |
| Leg agility – right leg | 1.96 | 0.364 | 1.31 | 1.4 |
| Leg agility – left leg | 1.79 | 1.37 | 1.32 |  |
| Arising from chair | 1.36 | 2.02 | 1.68 |  |
| Gait | 1.42 | 0.437 | 2.44 |  |
| Postural stability | 0.902 | 3.28 | 1.38 |  |
| Posture | 1.29 | 0.447 | 2.05 |  |
| Global spontaneity of movement | 2.76 | -0.172 | 1.1 | 1.22 |
